# Enhanced skin toxicity with concurrent ipilimumab and radiation in vaginal/vulvar melanoma: a case report and literature review

**DOI:** 10.1259/bjrcr.20160002

**Published:** 2016-07-29

**Authors:** Shane Mesko, Gottfried E Konecny, Paul C Tumeh, Mitchell Kamrava

**Affiliations:** ^1^School of Medicine, University of California, Irvine, Irvine, CA, USA; ^2^Department of Medical Oncology, University of California, Los Angeles, Los Angeles, CA, USA; ^3^Department of Medicine, University of California, Los Angeles, Los Angeles, CA, USA; ^4^Department of Radiation Oncology, University of California, Los Angeles, Los Angeles, CA, USA

## Abstract

Ipilimumab is a monoclonal cytotoxic T-lymphocyte-associated protein 4 antibody that has demonstrated improved survival in cutaneous melanoma. Little is known about the clinical impact of combining anti-cytotoxic T-lymphocyteassociated protein 4 therapy with radiation. Here we report a case of severe cutaneous desquamation in a 70-year-old female with vaginal/vulvar melanoma receiving concurrent ipilimumab and radiation therapy. The toxicity was successfully treated with oral/topical steroids and a break from treatment. This case underscores the importance of future research on optimal strategies for combining radiation with novel anti-tumour agents.

## Background

Vaginal/vulvar melanoma is a rare disease that is distinct in terms of natural history and underlying molecular pathogenesis when compared with cutaneous melanoma. Despite radical local surgical excision, almost 60% of vulvar melanomas and 80% of vaginal melanomas recur, highlighting the need to improve post-surgical outcomes.^1^ Ipilimumab is a monoclonal therapeutic antibody that blocks cytotoxic T-lymphocyte-associated protein 4 (CTLA-4) and has shown improved overall survival in cutaneous melanomas.^2^ However, little is known about the clinical impact of anti-CTLA-4 antibody in non-cutaneous melanoma types, or the safety and efficacy of combining anti-CTLA-4 antibody with local radiation. Here, we report a case of cutaneous drug eruption observed in a patient with vaginal/vulvar melanoma who was receiving combined ipilimumab and radiation in the recurrent setting.

## Case report

**Figure 1.**
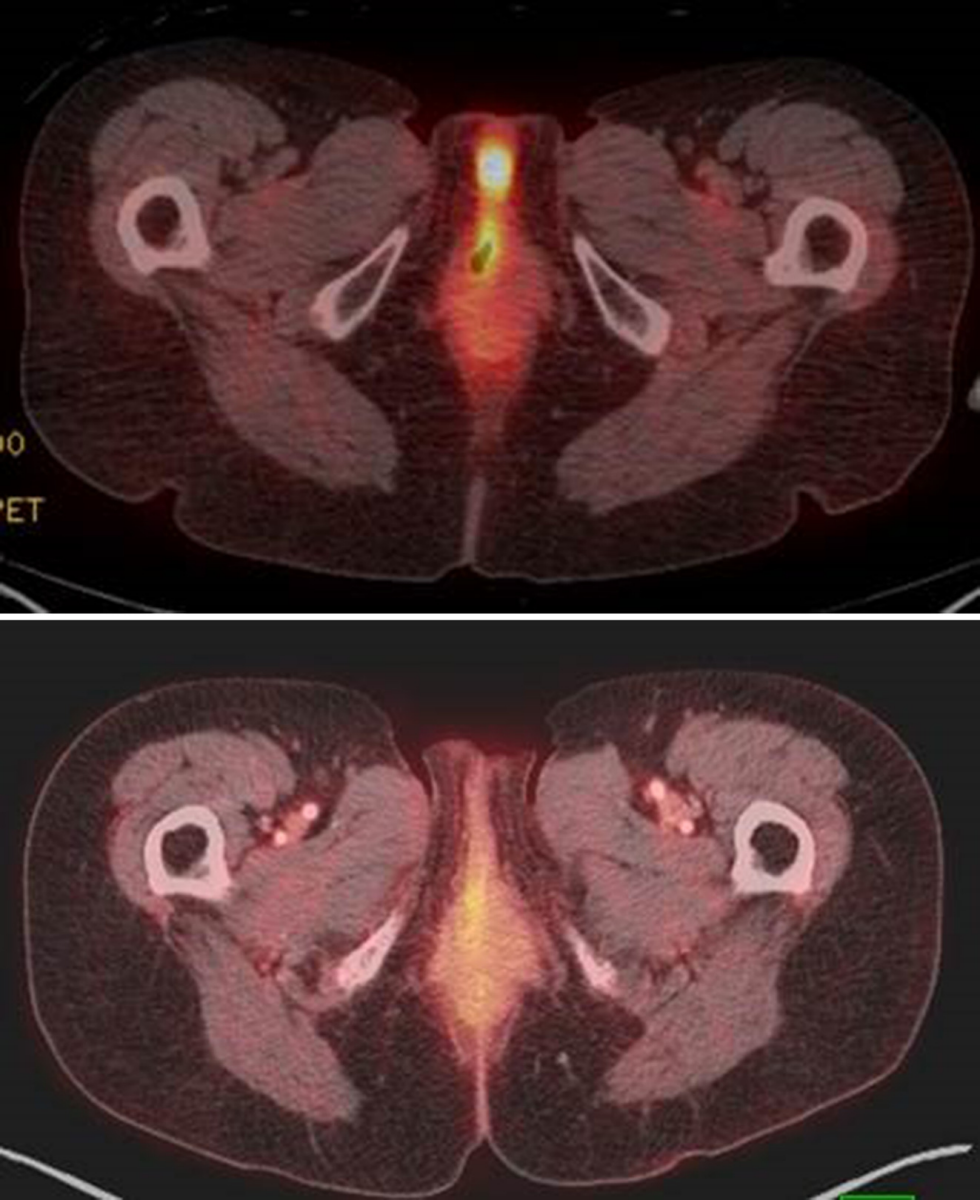
Top: Positron emission tomography/CT scan prior to the start of treatment, demonstrating a recurrent periclitoral mass. Bottom: complete resolution of the focal uptake at 15 months post treatment with radiation and ipilimumab.

A 70-year-old female with no significant past medical history, Eastern Cooperative Oncology Group performance status 0 and no prior incidence of hypersensitivity reactions, was incidentally found to have a small nodule in the proximal right posterolateral vaginal wall after presenting with post-menopausal bleeding. Subsequent biopsy demonstrated a 9 mm invasive melanoma and the patient underwent wide local excision with confirmed negative margins. 4 months later, she developed a right periclitoral mass. Positron emission tomography/CT scan at that time demonstrated focal uptake in this area but no regional/distant metastases (Figure 1). Excision demonstrated a large submucosal mass of atypical epithelioid cells with evidence of melanin synthesis, consistent with malignant melanoma. Breslow depth was 9 mm (3 mitoses/mm^2^) with a positive deep margin, and there was no evidence of lymphatic invasion. Her case was presented at a multidisciplinary tumour board and either additional surgery or radiation therapy was recommended to the patient. Owing to the significant morbidity anticipated with additional surgery, the patient opted for radiation therapy. Given the high risk of both local and regional/distant failure, concurrent chemotherapy was proposed. Owing to the historically poor response rates with standard chemotherapy, an immune pathway targeted agent was considered. This non-standard approach was actually initially proposed by the patient. After being thoroughly explained the current standard of care, in addition to the pros and cons of pursuing concurrent radiation and immunotherapy, the patient chose to proceed with combination immunotherapy and radiation treatment. Several studies have demonstrated that local radiotherapy primes and/or enhances an immune response through cytotoxic T lymphocytes.^3,4^ Concurrent immunotherapy may then further enhance the activity and/or duration of the downstream immune response.^5,6^ Given the historically low efficacy of our current treatment paradigms in this disease, as well as the preclinical/clinical rationale to combine radiation and immunotherapy, a strategy of pursuing a combination of ipilimumab with radiation was felt to be reasonable.

**Figure 2.**
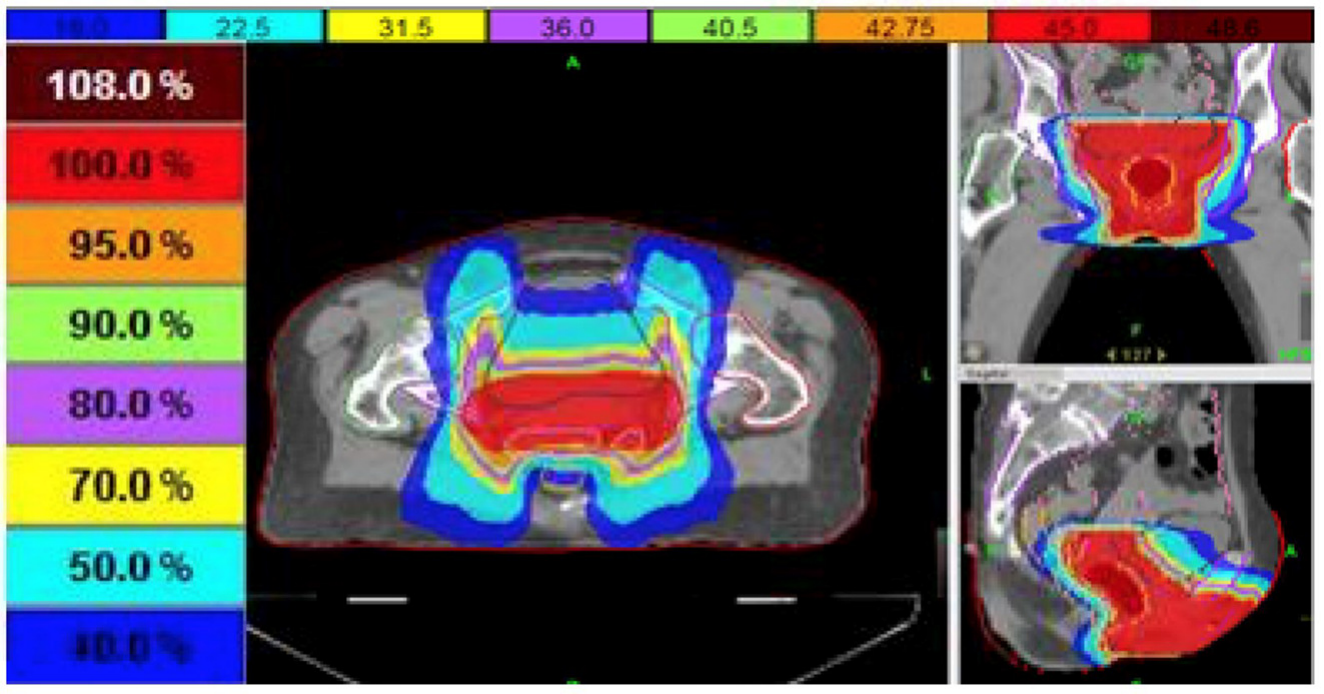
Intensity-modulated radiotherapy treatment plan showing dose distribution and treatment fields.

**Figure 3.**
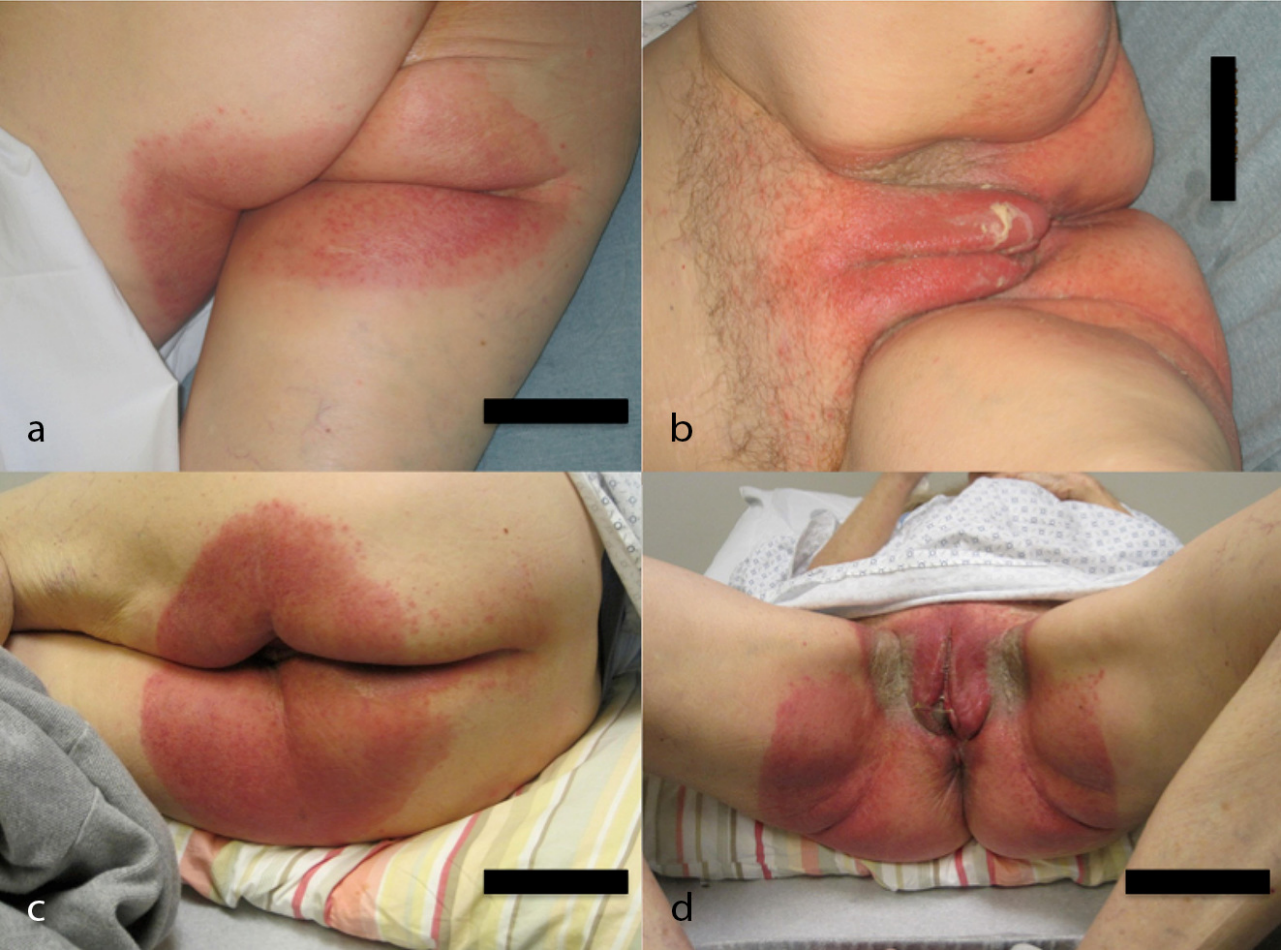
Grade 3 desquamating skin toxicity from concurrent ipilimumab and radiation, shown in the radiation field. (a,b) Initial demonstration of the reaction. (c,d) 1 day after the initial appearance.

**Figure 4.**
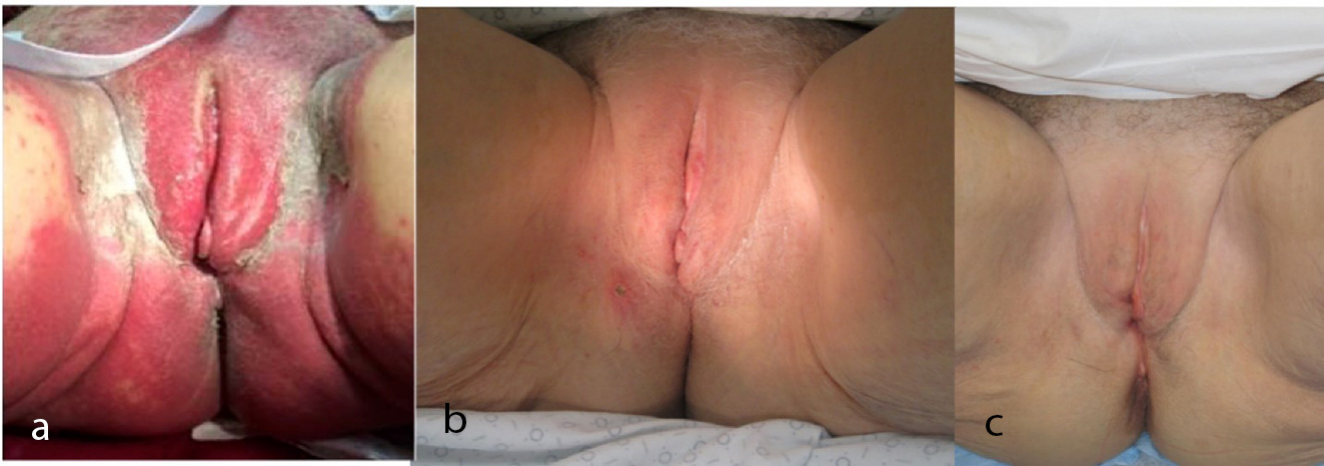
Photographs demonstrating temporal resolution of the reaction. (a) 10 days after the initial appearance. (b) 6 months post radiation completion. (c) 8 months post radiation completion. Treatment was with a combination of 0.1% topical triamcinolone, 60 mg oral prednisone and diphenhydramine for pruritus.

**Figure 5.**
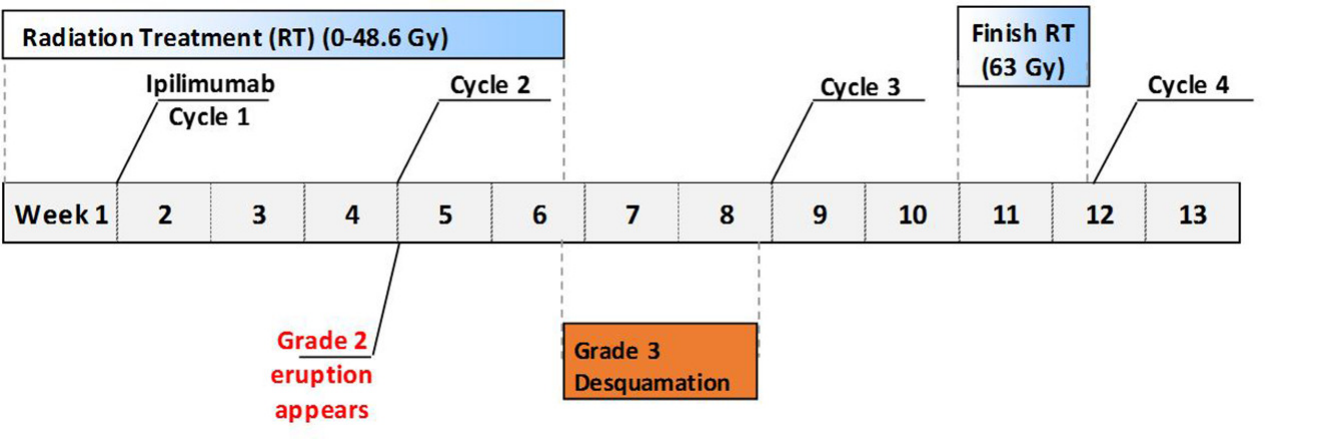
Timeline of events starting with the initial radiation treatment. Note that the skin reaction occurs 10 days after the second cycle of ipilimumab and 4 weeks after the first cycle. Treatment with oral and topical steroids led to resolution within 3 weeks.

Radiation was planned with intensity-modulated radiation therapy (IMRT) to the vulva and vagina (no elective nodal radiation to the groin) to an initial dose of 45 Gy (1.8 Gy/fraction over 25 fractions), and was to be followed by an electron boost to a total dose of 63 Gy (1.8 Gy/fraction over 10 fractions) at the site of the positive margin (Figure 2). A thermoluminescent dosimeter was placed on the vulva at the start of treatment to measure the skin dose and read as 1.78 Gy [95% confidence interval (1.66–1.90)], confirming that the planned dose was accurate on the skin. The patient received her first cycle of ipilimumab (3 mg kg^−1^) 7 days after the start of radiation, and the second cycle was delivered 3 weeks later when the patient was at a dose of 36 Gy. Around this time (3 weeks post ipilimumab cycle 1), she began to develop non-painful erythema in the vulvar and perianal area, as well as a pruritic, grade 2 cutaneous eruption that morphologically showed distinct erythematous papules that coalesced into thin plaques over the upper arms, chest, back and face/ears (all toxicities were graded using Common Terminology Criteria for Adverse Events version 4.03). She did not experience any fevers or other systemic symptoms. By 48.6 Gy dose (10 days post ipilimumab cycle 2), the patient developed a grade 3 skin reaction (Figure 3c,d) that was characterized as a moist desquamation with significant oedema, erythema and pain in the vaginal/vulvar/perianal region and was restricted to the radiation field (Figure 4a). A timeline of these events is illustrated in Figure 5. After proper consent, a 4 mm punch biopsy of the affected skin was performed and histopathological examination demonstrated spongiotic and interface dermatitis with a perivascular inflammatory infiltrate consisting of numerous eosinophils, consistent with a fixed drug eruption.

### Treatment

Radiation and ipilimumab were held given the severity of the moist desquamation, and the patient was referred to a dermatologist for evaluation of the ipilimumab-associated cutaneous eruption. It is noted that the patient had no existing risk factors or hypersensitivities predisposing her to an enhanced skin toxicity. The patient was started on 0.1% topical triamcinolone cream along with a methylprednisone dosepak. Given only mild improvement after 1 week, she was started on prednisone 60 mg daily (tapered over 7 days) with oral diphenhydramine for pruritus as needed. This resulted in significant improvement in her cutaneous eruption and pruritus. She then received a third cycle of ipilimumab (4 weeks after cycle 2) and resumed her periclitoral radiation boost without further issues after a 1-month break from radiation treatment. She went on to receive a fourth cycle of ipilimumab after completion of radiation without any complications.

### Follow-up

At follow-up 8 months post completion of radiotherapy, she had complete resolution of the in-field toxicity and improvement of her ipilimumab-associated cutaneous eruption (Figure 4b,c). Clinical examination and positron emission tomography/CT imaging 10 months after completion demonstrated no evidence of disease recurrence. Most recently, at her 15-month follow-up, she remains disease- and symptom-free (Figure 1).

### Patient perspective

At each follow-up visit, the patient has repeatedly expressed satisfaction with her decision to pursue immunotherapy rather than standard chemotherapy to complement her radiation treatment. She admits the initial side effects and intensity of the treatment course were difficult, although she is very pleased with the outcomes and would make the same decision if asked to do so again.

## Discussion

Cutaneous reactions with ipilimumab monotherapy represent the most common treatment-related side effect and have been well documented throughout the literature to affect as many as 75% of treated patients.^7,8^ It is most often described as an erythematous, oedematous rash with or without pruritus. It has been histologically characterized as a perivascular immune cell infiltrate with eosinophils and lymphocytes. The median time to onset of immune-mediated dermatitis is reported to be 3–4 weeks, with a range of up to 17.3 weeks.^9^ Management depends on severity, with grade 1 or 2 rashes being treated with topical steroids and oral antihistamine, and grade 3 reactions with oral steroids while holding ipilimumab. The temporal course and histological findings in our patient are highly consistent with this presentation.

Data on ipilimumab toxicity, whether it be skin or other adverse events, with concurrent radiation is much more limited. A retrospective analysis from Memorial Sloan Kettering Cancer Center on 29 patients treated with non-brain RT and ipilimumab found no significant difference in rates of adverse events with combination therapy when compared with single-agent ipilimumab.^10^ However, a trend of increased toxicity in patients receiving higher radiation doses was observed, including one grade 4 event in a patient being re-irradiated. Similarly, a recent case report showed an increase in the intensity and duration of a widespread maculopapular rash in a patient receiving 30 Gy (3 Gy/fraction) of palliative radiation and ipilimumab.^11^

The mechanisms underlying ipilimumab-associated cutaneous toxicity remain poorly defined.^8,12^ Even less is known about how radiation impacts this manifestation. It is important to consider that the existing data on combination therapy is limited primarily to patients receiving palliative doses of radiation. The relationship between cutaneous toxicity grade and higher doses of radiation combined with ipilimumab remain undefined. Radiosensitization and radiation-recall dermatitis are two potential explanations behind the observed reaction. Radiosensitization is typically described as an increased sensitivity to systemic agents when the increase in sensitivity after radiation occurs in < 7 days, while radiation recall is more often a later occurrence (> 7 days and up to 5 years later).^13^ Both are poorly understood and continue to be explored in the literature. The reaction in this patient does not conform well to either of these mechanisms given the concurrent nature of the treatments and lack of any further symptoms with resumption of therapy. It is difficult to discern whether the ipilimumab, radiation or simply the combination of the two primarily contributed to the cutaneous toxicities mentioned in this report.

We believe that the severity of the skin reaction seen at around 48.6 Gy of radiation is greater than what we would typically expect, as no grade 3 skin toxicities were noted when treating to a dose of 46.4 Gy with concurrent chemotherapy to the vulva using a similar radiation technique (IMRT).^14^ Other series using IMRT to the vulva have demonstrated similar findings; however, grade 3 reactions may be more prevalent when using older three-dimensional conformal radiation techniques.^15^ There is currently an open clinical trial in cervical cancer looking at adding ipilimumab in the adjuvant setting after definitive chemotherapy and radiation, and there have been no significant skin reactions seen using this sequential approach [Jyaoti Mayadev, 2016, personal communication].^16^

Limitations of this case include it being a report representing the experience of one patient at a single institution. Thus, this reaction may potentially not be generalizable to a larger population of patients. However, as combinations of immunotherapy and radiotherapy continue to be explored in the literature, it is important to document any unexpected findings.

## Conclusions

This case of vaginal/vulvar melanoma demonstrates the potential for increased cutaneous skin reactions when combining radiation and ipilimumab. It remains difficult to conclude whether the severity of the response was owing to the combination therapy or to a patient-specific sensitivity to one of the therapies. However, with the emergence of a wide variety of combinatorial strategies, the impact of radiation therapy on the tumour microenvironment will be critical to understanding which radiation strategy and combination checkpoint inhibitor will be best suited for a specific patient. Further investigation through prospective studies will better define this risk.

## Learning points

1. Caution should be exercised when combining radiation and ipilimumab, as there may be potential for increased cutaneous skin reactions.
2. Oral/topical steroids with a break in treatment resulted in full resolution, and re-initiation of both ipilimumab and radiation did not result in further reactions.
3. As novel anti-tumour agents continue to emerge, developing a better understanding of their interaction with radiation will be critical in determining the optimal combination of available therapies.

## Consent

Written informed consent for the case to be published (including images, case history, and data) was obtained from the patient for publication of this case report.
